# Elastography for hepato-biliary-pancreatic surgery

**DOI:** 10.1007/s00595-013-0799-7

**Published:** 2013-11-30

**Authors:** Yosuke Inoue, Norihiro Kokudo

**Affiliations:** 1 Department of Gastrointestinal Surgery, Cancer Institute Hospital, Japanese Foundation for Cancer Research, Tokyo, Japan; 2 Department of Hepato-Biliary-Pancreatic Surgery and Artificial Organ and Transplantation Division, Graduate School of Medicine, University of Tokyo, Tokyo, Japan

**Keywords:** Elastography, Liver, Intraoperative diagnosis, Palpation

## Abstract

Palpation is a subjective and non-sharable diagnostic method. Recently, palpation has been supported and replaced by elastography, which provides a novel parameter of “stiffness” as a visual representation or quantified value. Today, elastography is performed using two major modalities: strain elastography and shear wave elastography. Strain elastography converts the extent of deformation during external compression into colors, displaying these colors as a strain map in a motion picture representing the relative elasticity inside the region of interest. Shear wave elastography can quantify the elasticity of a target by calculating the velocity of shear waves generated by a probe. In addition to superficial organs, elastography has also been applied to upper abdominal organs, including the liver, pancreas and spleen. The visualization of the stiffness of focal lesions in the liver or the pancreas has enabled a more sensitive and specific depiction of small, non-palpable nodules, which are difficult to depict using B-mode ultrasonography. The quantification of stiffness also enables non-invasive estimates of liver fibrosis, the risk of postoperative liver insufficiency and the risk of recurrence of viral hepatitis after transplantation. In this article, we review the major reports that have recently been published describing the effective application of elastography to solid upper abdominal organs in a clinical setting.

## Introduction

Elastography is a method used to visualize and quantify the stiffness of an object. Palpation of tissues and organs, especially the abdominal organs, has long been performed using an examiner’s hands, but as the results of such examinations cannot be shared with another person, this method lacks objectivity. Elastography enables information regarding the stiffness to be shared as a visual representation or quantified value among physicians, providing a more objective parameter than manual palpation.

Ultrasonography has long been used as a noninvasive and rapid diagnostic modality for solid organs in the upper abdomen, including the liver [1, 2] and pancreas [3, 4]. During open surgery for these organs, manual palpation plays an important role in the intraoperative diagnosis and identification of lesions within these organs [5]. Although upper abdominal solid organs should be good candidates for elastography, this method has seldom been applied to these organs, compared with its more prevalent use for superficial organs such as the breast [6, 7], thyroid [8, 9] and prostate [10, 11]. There are two main reasons for this. The first is the location, in that abdominal organs are located at a distance from the body surface, and obstacles such as muscles, fascia, fat and skin are present, instead of a stable base. Furthermore, these organs are easily affected by heart motion or breathing. The second reason is the technical limitation of traditional elastography, in which homogenous, axial and trace compression are required to obtain optimal elastic images. Because of these difficulties, the first report describing the application of elastography for focal lesions in solid abdominal organs involved an elastography device mounted on an endoscopic ultrasound (EUS) probe, which allowed the probe to be applied almost directly to the organ surface [12]. Subsequently, many investigators have developed new methods for applying probes or other devices for elastography, and the number of reports regarding elastography for abdominal organs has been increasing rapidly.

We herein review the major articles regarding the application of elastography to solid upper abdominal organs and consider the future of this modality and its use in upper abdominal surgery.

## Methods used for elastography

The term “elasticity” indicates the physical property of materials that deform reversibly under compression. In general, the harder or stiffer an object is, the less elasticity it has. This term should be used to express a quality of the subject and is not a quantified value. When one expresses the quantified value, the term “stiffness” is more appropriate.

**Fig. 1:**
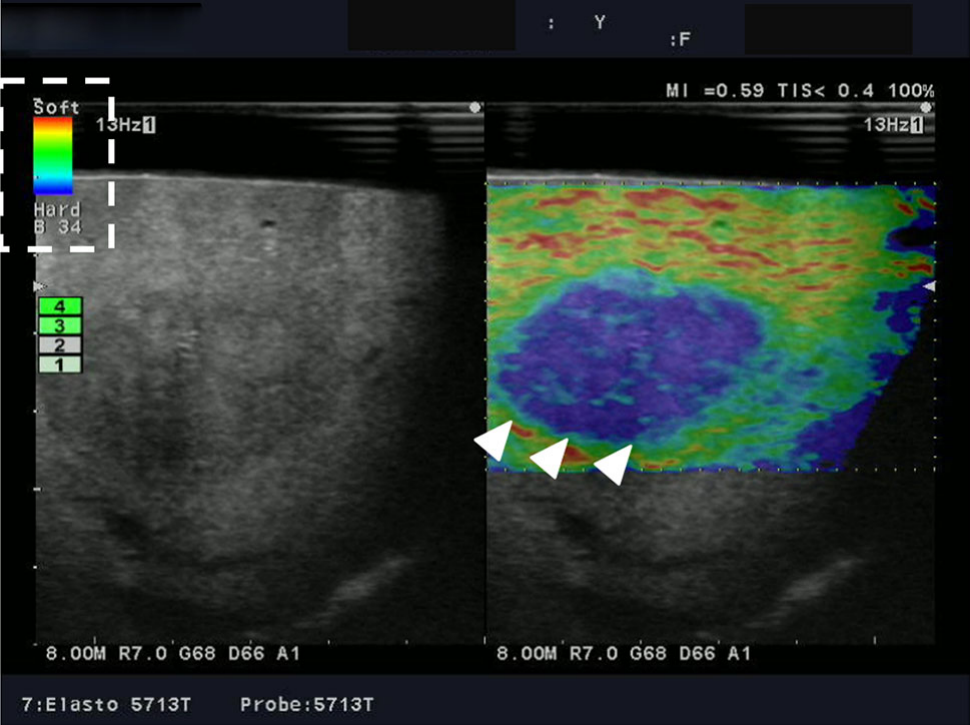
Real-time tissue elastography intraoperatively applied directly to the liver surface. The elastographic image is displayed for the area corresponding to the ROI in the B-mode image. A clearly demarcated bluish area is depicted, indicating a hard tumor (*white arrowhead*). The degree of stiffness can be evaluated based on the color scale displayed in the upper left side (*dotted box*)

There are two types of elastography: strain elastography (SE) and shear wave elastography (SWE). SE measures the extent of deformation of the subject during external compression using ultrasonography [13] and converts the deformation of each particle inside the ROI into a specific color [14]. For example, in real-time tissue elastography (RTE; Hitachi-Akola, Japan), harder areas with minimum deformation are expressed as blue, while softer areas with larger deformation are expressed as yellow to red colors [14]. The probe should be applied to the surface of the organ axially with only a light pressure, since the association between the pressure and strain is essentially proportional. When applied to the liver externally or intraoperatively, an elastogram can be obtained from not only intentional compression, but also from trace displacements arising from the heart beat (Fig. 1). Using this method, nodules with different elasticity can be accurately identified within a homogenous background, such as the liver tissue, even if the nodule has the same echogenicity as the background when examined using B-mode US [12, 15–17].

**Fig. 2:**
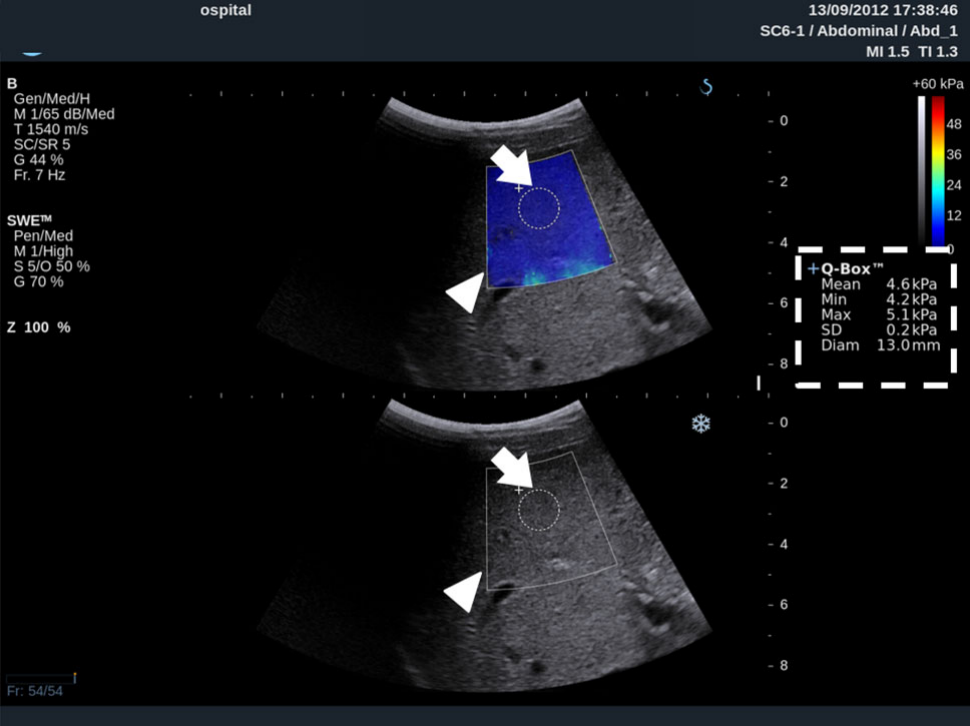
Shear wave elastography (supersonic shear imaging) applied externally to the liver. An elasticity map is displayed for the area corresponding to the ROI (*white arrowhead*) in the B-mode image shown below. Another ROI, called the “Q-Box” (*white arrow*), with an arbitrary size can be set to calculate the average value of the stiffness within the area (kPa). The calculated score is displayed on the right side (*dotted box*)

Shear wave elastography is distinct from the strain method. This modality calculates the velocity of the propagation of shear waves inside the subject [18, 19]. Shear waves are low-frequency waves that are generated by mechanical knocks with the probe [20] or by acoustic impulses [21]. This method can visualize not only the distribution of stiffness, but can also quantify the stiffness of the subject. Moreover, the method does not require any direct touching or compression. This method can be used to evaluate deeply located targets and areas beyond intervening fluid, which SE has difficulty depicting (Fig. 2).

## Application to abdominal organs

Compared with surface organs, such as the breast, superficial vessels or the thyroid, providing adequate compression and obtaining a clear image can be difficult when examining intraabdominal organs, especially using strain imaging. One possible solution for the adequate application of elastography to abdominal organs is the application during abdominal surgery or endoscopy [12, 15, 16, 22–26]. Such applications would enable direct contact between the probe and the organ surface, which would provide a clearer image than that obtained through the abdominal wall, and would also enable appropriate compression of the organ to be manually regulated. Intraoperative US is regarded as an essential diagnostic modality for hepato-biliary-pancreatic surgery [2, 27], and intraoperative or laparoscopic US probes are commonly available in high volume centers.

Another possibility for the robust application of elastography to abdominal organs is the use of SWE. This modality can allow the measurement of stiffness at depths as far as radiofrequency signals are able to propagate into the subject, even if fluid exists between the probe and the target area. This ability is a distinct advantage of SWE, and together with its ability to provide quantifiable information, has led to a rapid increase in the number of reports published regarding the use of SWE to examine abdominal organs.

## Elastography for the liver

The first clinical report of elastography for the liver was based on the application of transient elastography (TE, Fibroscan, Echosens, France), which was the first generation of SWE, for the purpose of estimating the extent of liver fibrosis [20, 28, 29]. TE utilizes a mechanical knock to create shear waves that are generated from the probe itself, and the shear wave velocity is then measured using the same probe [20]. The method converts the velocity of the shear wave to a representation of the stiffness of the target (kPa). Although the gold standard for the diagnosis of liver fibrosis had previously been a needle biopsy, this method was sometimes unreliable because of sampling errors or discrepant findings at multiple puncture sites and also carried a risk of complications, such as bleeding or injury to other organs [30–33]. These limitations created a demand for a noninvasive and reliable method of evaluating liver fibrosis. Estimates of fibrosis based on a serological algorithm or MRI elastography have been reported, but the number of articles on TE has now exceeded 600, and this application of TE has been widely disseminated in clinical settings, mainly in Western countries. Although TE is indeed a promising modality, it has some limitations when applied for purposes other than estimating fibrosis. For example, the TE probe is not equipped with a B-mode ultrasonography guide, which is essential to set the region of interest (ROI) appropriately, especially in obese patients or postoperative patients [34–36]. This limitation has precluded its dissemination in the field of liver surgery, where patients often have heterogeneous liver components because of the presence of neoplasms, previous resections or transplanted liver tissue.

Recently, a novel SWE method has been developed in which shear waves are generated as strong and low-frequency acoustic pulses, and the propagation velocity of these waves is evaluated using ultrasonography. With this second-generation method, the ROI can be arbitrarily set within the ultrasonography field, and the stiffness inside the ROI can be estimated as an absolute value expressed in terms of the propagation velocity (m/s) (acoustic radiation force impulse, ARFI; Siemens, Germany) [21, 37]. Alternatively, a stiffness map can be displayed on the monitor, and the mean stiffness value (kPa) of the ROI, the size and location of which can be arbitrarily set, can be calculated (supersonic shear imaging, SSI; Supersonic Imaging, France) [38, 39].

**Fig. 3:**
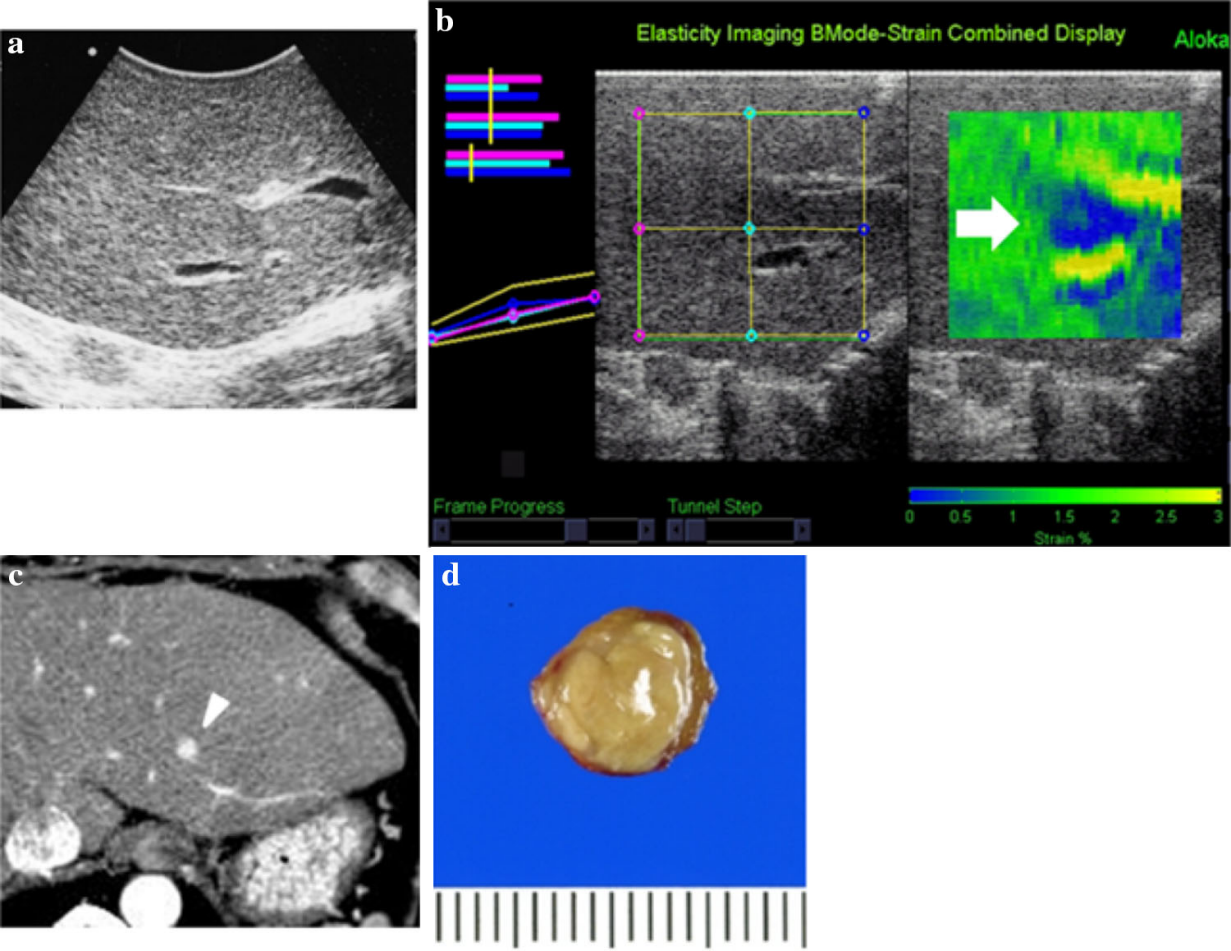
(Reproduced from Ref. [15]): Hepatocellular carcinoma in segment 2. **a** B-mode IOUS failed to identify a small HCC, partly because of its isoechoic content, the lack of a halo sign and the adjacent vascular structures. **b** Preoperative CT showed a small nodule enhanced in the arterial phase (*white arrowhead*). **c** Elastography depicted a clearly demarcated blue region (*white arrow*) just above the portal vein in segment 2, indicating the existence of a hard nodule. **d** The resected specimen. The nodule proved to be an HCC with a capsule

In the field of liver surgery, elastography was first reported as an intraoperative diagnostic modality used to evaluate focal liver lesions [16]. Before the introduction of elastography, liver surgeons knew that most liver tumors had different degrees of stiffness relative to the background liver parenchyma. However, a subjective description of tumor stiffness had long been impossible. An attempt to depict liver lesions using elastography was initiated in 2007 using RTE, a form of SE [12]. To apply adequate compression to the target tissue and to obtain a clear elastogram, the number of obstacles between the liver surface and the US probe should be minimized as much as possible. Consequently, the RTE series employed mini-probes for intraoperative use or for with a EUS probe. In particular, intraoperative applications of RTE were very useful for depicting lesions that were difficult to identify using external elastography (Fig. 3). Moreover, intraoperative elastography showed a better diagnostic performance than B-mode ultrasonography [15].

During open surgery, one can apply mini probes from various directions after the mobilization of the liver, allowing for a depiction of deeply located lesions that cannot be depicted using external applications. Since surgeons can perform manual palpation as needed during open surgery, elastography is advantageous in a limited number of subjects, such as those with deeply located, tiny lesions or those in whom the lesions appear as iso-echoic areas using B-mode US. In the setting of laparoscopic liver resection, however, manual palpation of the liver is difficult, and elastography may play an important role as a form of “virtual palpation.”

Since RTE does not quantify the absolute stiffness value, but instead visualizes the elastic contrast between the lesion and the background, its results might be less robust for individuals with heterogenic backgrounds, such as those with fibrotic or cirrhotic livers. The intraoperative use of ARFI or SSI is desired, and the development of intraoperative probes or laparoscopic probes should be promoted.

Novel uses for elastography in the field of liver surgery were identified in a recent article assessing liver function using an elastic parameter [40–44]. Kim et al. [40] first applied SWE using TE to predict postoperative liver insufficiency and showed that the liver stiffness was the only independent risk factor predicting postoperative liver insufficiency, indicating that elastography is promising as a preoperative risk indicator for liver resection. This indication was supported by several additional larger series using TE [42, 43]. Harada et al. [44] reported the efficacy of ARFI estimations of the preoperative liver stiffness for predicting postoperative complications, including recurrent ascites, after liver resection. To date, the liver functional reserve has been assessed using the indocyanine green retention test, the Child-Pugh classification [45, 46] or GSA scintigraphy [47–49]. An examination of liver stiffness is easy and non-invasive, and has the potential to be useful as another supportive modality for estimating the liver function.

## Elastography for liver transplantation

In the field of liver transplantation, measuring the stiffness of the transplanted liver tissue can provide useful information. For example, elastography has been reported to be useful for predicting the recurrence of hepatitis C virus (HCV) after transplantation in patients with HCV-related liver disease [50–53]. For the diagnosis of HCV remission after liver transplantation, a needle biopsy has been regarded as the gold standard, although this procedure is associated with a risk of complications such as bleeding or injury to other organs, especially in patients who have impaired clotting function or are undergoing anticoagulant therapy after liver transplantation [54]. Noninvasive elastography could, therefore, replace biopsies in screening for HCV remission or for making decisions regarding interferon treatment.

Moreover, acute cellular rejection after transplantation can be predicted or diagnosed noninvasively using elastography. Acute cellular rejection can occur during acute periods after transplantation, when invasive examinations such as a needle biopsy should be avoided due to concerns about fatal bleeding in patients receiving post-transplant anticoagulant therapy [54]. Inoue et al. [35] documented changes in the stiffness of a liver graft after transplantation, in which the graft rapidly stiffened during the first week and then gradually softened toward a normal stiffness over the course of 5 weeks. In a case of significant acute rejection, the graft stiffness increased again, and this was accompanied by a decrease in the portal flow. Although acute cellular rejection (ACR) has long been diagnosed based on a liver biopsy, elastography may play a contributory role for predicting or evaluating ACR. In support of this, Yoon et al. [55] used SSI to examine post-transplant livers, and advocated its efficacy for predicting the presence of rejection or recurrent hepatitis during post-liver transplantation (LT) follow-up examinations, especially those performed more than 4 weeks after transplantation.

## Elastography for the pancreas

The pancreas could be a promising candidate target for elastography. The radiological findings of pancreatic tumors vary widely, and decision making for surgery is sometimes very difficult, especially when the lesion mimics a benign tumor or localized pancreatitis. A histological diagnosis can be made using EUS fine needle aspiration cytology [56, 57]; however, this invasive modality is associated with risks of cancer dissemination and serious complications [58–61]. Since palpation of the pancreas from the body surface is difficult, preoperative information obtained using elastography regarding the stiffness of a pancreatic tumor could be useful.

To date, reports on the use of elastography to examine the pancreas have mainly utilized EUS-RTE, in which the probe is nearly directly applied to the pancreas. The main goal of these studies was the differentiation of invasive ductal cancer from benign lesions, such as chronic pancreatitis or mass-forming pancreatitis. Itokawa et al. [62] used EUS-RTE to examine 109 patients with pancreatic diseases and demonstrated that the mean strain ratio (non-mass area/mass area) of cancers was significantly higher than that of mass-forming pancreatitis. They reported a success rate of 100 % for obtaining EUS-RTE images. On the other hand, Hirche reported a rather lower success rate for EUS-RTE (56 % of patients with solid pancreatic lesions), and the main reasons for the incomplete acquisitions were poor delineation of the border of lesions greater than 35 mm in diameter (39 %) or of lesions at a larger distance from the transducer (10 %) [23]. Since large pancreatic lesions can be easily identified or differentiated without using RTE, this modality is likely to be most useful for the differentiation and delineation of tiny lesions. Indeed, Hirche reported that the majority of lesions smaller than 35 mm in diameter were adequately and reproducibly evaluated using EUS-RTE (91 %).

SWE has also been increasingly applied to examinations of the pancreas, mainly using an external method. Yashima et al. applied ARFI to 46 patients with chronic pancreatitis and 52 healthy volunteers and showed the patients with chronic pancreatitis had a significantly harder pancreas than those with a normal pancreas. The area under the ROC curve was 0.78, with an optimal cut-off value of 1.40 m/s [63].

In the field of pancreatic surgery, the stiffness of the pancreas is known to be significantly related to the risk of postoperative pancreatic fistula formation [49, 64, 65]. Although reports comparing pancreatic stiffness and the incidence of postoperative pancreatic fistulas have never been published, quantification of the stiffness of the pancreatic parenchyma could provide insight into the risk of pancreatic fistula formation.

## Elastography for the spleen

The spleen is a solid organ located in the upper left edge of the abdomen and is accessible for external elastography. Splenic tumors are rare, and their diagnosis is unlikely to be a common problem. On the other hand, the splenic stiffness has been reported to be useful for estimating the degree of liver cirrhosis and the risk of esophageal varices [66–68]. Colecchia et al. [70] conducted stiffness measurements of the spleen and liver in 100 patients with hepatitis HCV-related cirrhosis and compared its performance for predicting portal hypertension (PH) and esophageal varices (EV) with other parameters, including the hepatic vein pressure gradient and noninvasive scores of the PH and EV, such as the platelet count/spleen diameter ratio [69] and the liver stiffness—spleen diameter to platelet ratio score. They concluded that the spleen and liver stiffness measurements were more accurate than the other parameters and advocated their use as a noninvasive assessment and for the monitoring of the PH and EV.

These findings suggest that splenic stiffness, with or without liver stiffness, might be a useful parameter for estimating the perioperative liver function. Overman et al. [71] reported that splenic volume changes were significantly related to the extent of sinusoidal injury after oxaliplatin-based chemotherapy. If this is the case, the splenic stiffness should change after chemotherapy, and monitoring the changes in stiffness using elastography could provide a noninvasive and convenient means of predicting the liver function and would be a novel criterion for assessing the liver functional reserve prior to post-chemotherapy liver resection.

## Elastography for the biliary system

Since the biliary system is not characterized by a solid organ, the effective application of elastography in this field has been limited. Kapoor et al. conducted a prospective trial of elastographic evaluations of gallbladder wall thickening using ARFI. They reported a diagnostic performance that had a sensitivity and specificity of 100 and 91.3 %, respectively, and the cutoff value for diagnosing gallbladder carcinoma based on the mean shear wave velocity was 3.41 m/s (*P* < 0.0001), and the area under the curve was 0.92. They concluded that elastography was a promising modality for differentiating between benign and malignant gallbladder wall thickening and should be used in combination with B-mode US [72]. In other biliary diseases, elastography has been reported to be useful as a noninvasive marker for primary biliary cirrhosis or the prediction of upper GI varices in children with biliary atresia [73–80].

## Comments

We have reviewed articles regarding the application of elastography to solid upper abdominal organs. In the fields of gastroenterology and hepatology, elastography has become one option that can be used as a substitute for traditional invasive examinations, such as needle biopsy, gastrointestinal endoscopy and the measurement of the hepatic venous pressure gradient.

On the other hand, the dissemination of elastography in the fields of hepatobiliary-pancreatic surgery and laparoscopic surgery remains limited. Ultrasonography and palpation are, however, essential tools in this field, and a trend toward less invasive surgery, including laparoscopic or robotic surgery, has the disadvantage of not enabling palpation. Although both laparoscopic and/or robotic surgery have been regarded as superior methods due to their low-invasiveness [81], the lack of palpation can lead to a deterioration of the intraoperative diagnostic performance and could preclude future innovations in this type of surgery. Attempts to apply elastography during laparoscopic surgery have been initiated, and the prospective accumulation of experience regarding “virtual palpation” as a substitute for manual palpation is needed.

In conclusion, elastography is a promising diagnostic modality that provides a novel parameter of objective stiffness and could have a major impact on the future of surgery. The prompt development of methods for the effective application of elastography to hepatobiliary-pancreatic surgery and laparoscopic surgery is urgently needed for further innovation in this field.

## Abbreviations

EUS: Endoscopic ultrasound
SE: Strain elastography
SWE: Shear wave elastography
RTE: Real-time tissue elastography
TE: Transient elastography
ROI: Region of interest
ARFI: Acoustic radiation force impulse
SSI: Supersonic shear imaging
EV: Esophageal varices
PH: Portal hypertension
